# Prevalence of depression and validation of the Beck Depression Inventory-II and the Children's Depression Inventory-Short amongst HIV-positive adolescents in Malawi

**DOI:** 10.7448/IAS.17.1.18965

**Published:** 2014-07-30

**Authors:** Maria H Kim, Alick C Mazenga, Akash Devandra, Saeed Ahmed, Peter N Kazembe, Xiaoying Yu, Chi Nguyen, Carla Sharp

**Affiliations:** 1 Baylor College of Medicine International Paediatric AIDS Initiative, Texas Children's Hospital, Houston, TX, USA; 2 Baylor College of Medicine–Abbott Fund Children's Clinical Centre of Excellence, Lilongwe, Malawi; 3 Department of Paediatrics, Epidemiology Center, Baylor College of Medicine, Houston, TX, USA; 4 Department of Psychology, University of Houston, Houston, TX, USA

**Keywords:** HIV, adolescents, depression, prevalence, BDI-II, CDI-II-Short

## Abstract

**Introduction:**

There is a remarkable dearth of evidence on mental illness in adolescents living with HIV/AIDS, particularly in the African setting. Furthermore, there are few studies in sub-Saharan Africa validating the psychometric properties of diagnostic and screening tools for depression amongst adolescents. The primary aim of this cross-sectional study was to estimate the prevalence of depression amongst a sample of HIV-positive adolescents in Malawi. The secondary aim was to develop culturally adapted Chichewa versions of the Beck Depression Inventory-II (BDI-II) and Children's Depression Inventory-II-Short (CDI-II-S) and conduct a psychometric evaluation of these measures by evaluating their performance against a structured depression assessment using the Children's Rating Scale, Revised (CDRS-R).

**Study design:**

Cross-sectional study.

**Methods:**

We enrolled 562 adolescents, 12–18 years of age from two large public HIV clinics in central and southern Malawi. Participants completed two self-reports, the BDI-II and CDI-II-S, followed by administration of the CDRS-R by trained clinicians. Sensitivity, specificity and positive and negative predictive values for various BDI-II and CDI-II-S cut-off scores were calculated with receiver operating characteristics analysis. The area under the curve (AUC) was also calculated. Internal consistency was measured by standardized Cronbach's alpha coefficient, and correlation between self-reports and CDRS-R by Spearman's correlation.

**Results:**

Prevalence of depression as measured by the CDRS-R was 18.9%. Suicidal ideation was expressed by 7.1% (40) using the BDI-II. The AUC for the BDI-II was 0.82 (95% CI 0.78–0.89) and for the CDI-II-S was 0.75 (95% CI 0.70–0.80). A score of ≥13 in BDI-II achieved sensitivity of >80%, and a score of ≥17 had a specificity of >80%. The Cronbach's alpha was 0.80 (BDI-II) and 0.66 (CDI-II-S). The correlation between the BDI-II and CDRS-R was 0.42 (*p*<0.001) and between the CDI-II-S and CDRS-R was 0.37 (*p*<0.001).

**Conclusions:**

This study demonstrates that the BDI-II has sound psychometric properties in an outpatient setting among HIV-positive adolescents in Malawi. The high prevalence of depression amongst HIV-positive Malawian adolescents noted in this study underscores the need for the development of comprehensive services for HIV-positive adolescents.

## Introduction

In 2009, an estimated 5 million young people (aged 15–24) and 2 million adolescents (aged 10–19) were living with HIV, the vast majority in sub-Saharan Africa [1]. Nearly one in two new HIV infections occurs in young people [1]. This, combined with the successful scale-up of paediatric HIV services resulting in improved survival into the teen years, has led to a rising demand for comprehensive services focusing on the special needs of adolescents [2].

Adolescence is a period of vulnerability for a host of well-documented biological, behavioural, social and structural reasons. Adolescents living with HIV/AIDS in sub-Saharan Africa present unique challenges to health care providers [3,4]. Furthermore, mental health in people living with HIV and AIDS (PWLHA) is an area of scanty research, and there is a dearth of evidence for adolescents, particularly in the African setting.

Depression is a major contributor to the burden of disease worldwide and is estimated to be the leading cause of disability as measured by Years Lost due to Disability (YLDs) [5,6]. The prevalence of depression is estimated to be higher in developing versus developed countries [7]. In PLWHA, the prevalence of depression has been documented to be as high as double that of the general population [8]. In the few studies in Africa, estimates of prevalence of depression in PLWHA range between 12 and 60% [8–14].

Depression has been shown to worsen several HIV-related health outcomes. It is associated with steeper declines in CD4 counts, and more rapid progression to AIDS and death [8,14–16]. Associations with suboptimal antiretroviral therapy (ART) adherence and discontinuation have been reported with significant implications for long-term treatment efficacy [17–19]. Depression in youth has also been correlated with high-risk behaviour including earlier sexual debut, low condom use, substance abuse, more frequent sexual partners and unplanned pregnancy [11,20]. However, most evidence comes from high-income countries, and few associations have been firmly established in sub-Saharan Africa, the epicentre of the HIV epidemic. Other correlates more applicable to Africa and the Malawian setting in particular, such as orphanhood, poverty and urban migration, have been poorly described.

The lack of clinical data, limited awareness by healthcare providers and patients and the scarcity of resources and interventions, all act as obstacles in the provision of holistic care to adolescents living with HIV. There is an urgency to incorporate mental health into adolescent HIV care in Malawi and sub-Saharan Africa to improve quality of life and health outcomes.

Unfortunately, there are few studies in sub-Saharan Africa, and virtually no published studies in Malawi validating the psychometric properties of the commonly utilized diagnostic and screening tools for depression amongst adolescents as compared to adults [13,21–24]. Depression may manifest in a variety of ways across different cultural and age groups, and research instruments need to be culturally appropriate [25]. Traditional instruments developed in the west should, if used, undergo a careful process of translation, back-translation and modification to ensure cross-cultural equivalence [21,25].

The Beck Depression Inventory-II (BDI-II) and the Children's Depression Inventory-II-Short (CDI-II-S) were used as depression screening tools. The BDI-II is a 21-item tool that has been extensively tested for validity and reliability since the 1960s [26], including use in the paediatric population. One study in Nigeria provided psychometric evidence in support of the BDI-II in the African setting [22]. The CDI-II-S, a symptom-oriented instrument, is a 12-item self-report tool used for assessing depression in children aged 7–17 years. Psychometric evidence in support of the use of the long version was provided in a study amongst children in Tanzania [13]. The CDI-II-S has been validated as being equally efficacious for screening purposes as the long version [27–29]. Both tools can be completed in 5–10 minutes and, therefore, are ideal for use in typical high-volume clinical settings in Malawi. There has been no assessment of either tool in Malawi to date.

Malawi, a land-locked country located in Southern Africa, with an HIV prevalence of 11%, has successfully scaled up access to HIV treatment services with 276,987 patients retained alive on ART as of June 2011 [30]. However, there are limited local options for diagnosis and treatment of mental health problems. One previous survey of depression in a cohort of orphaned children and adolescents (10–18 years) in the southern region reported a 53.2% prevalence for clinical depression using the Center for Epidemiological Studies Depression Scale Modified for Children (CES-DC) [31].

The primary aim of this cross-sectional study was to estimate the prevalence of depression amongst a sample of HIV-positive adolescents in Malawi. The secondary aim was to develop culturally adapted Chichewa (the official and most widely spoken language in Malawi) versions of the BDI-II and CDI-II-S and conduct a psychometric evaluation of these measures for use in HIV-positive adolescents by evaluating their performance against a structured depression assessment using the Children's Depression Rating Scale, Revised (CDRS-R).

## Methods

### Study population

We recruited a convenience sample of adolescents aged 12–18 years from the Baylor College of Medicine Children's Clinical Center of Excellence (COE) and Zomba ART Clinic. The COE, the largest and first stand-alone paediatric HIV clinic in Malawi, is located in Lilongwe, in central Malawi. It serves both as an outpatient facility and as a national referral centre for paediatric HIV care. Patients at both sites come from urban and rural locations and are of various ethnic and socio-economic backgrounds. As of March 2011, there were >420 (COE) and >200 (Zomba) patients 12–18 years of age active in care.

### Informed consent

The National Health Sciences Research Committee (NHSRC) in Malawi and Baylor College of Medicine Institutional Review Board in USA approved the study protocol. The COE and Zomba Central Hospital granted site approvals. All caregivers and adolescents signed written informed consents/assents.

### Measures

The CDI-II-S and the BDI-II were selected as the most appropriate tools to translate and evaluate for use in assessing depression amongst Malawian adolescents living with HIV. Both tools are widely used and validated self-report measures of depression in youth.

The CDI-II-S is a 12-item self-report instrument used to detect the presence and severity of depressive symptoms in children aged 7–17 years [28,29]. The reading level is rated to be at the 2nd grade, and it can be completed in 5–10 minutes [28,29]. Each item is scored: 0 for no symptom; 1 for a mild; and 2 for definite symptoms [13,29]. The CDI-II-Long [29,32–34] from which the CDI-II-S is derived has been validated in a number of countries including Tanzania [13,35–37]. The CDI-II-S is reported to be a psychometrically comparable screening instrument for depression [27–29]. Therefore, the CDI-II-S was selected as the measure most appropriate to translate and examine for use in typical high-volume clinical settings in Malawi.

The BDI-II is a 21-item self-report questionnaire that assesses the presence and severity of depressive symptoms in adolescents ≥13 years [26,35,38]. Each question is scored: 0=symptom absent; 1=symptom present; 2=moderate symptom; and 3=severe symptom [35,39]. Total potential score is 63. The reading level is rated to be at the 6th grade, and it can be completed in about 10 minutes. The tool has been validated in other countries where English is not the primary language [22,35,38].

To achieve accuracy in translation and cultural understanding, the tools underwent an adaptation process. The process integrated input from multiple collaborators including US, UK, Malawi-based researchers, linguistic experts and Malawian adolescents. The tools were translated into Chichewa by linguistic experts from Mzuzu University Centre for Language Studies, back-translated, and edited. To help ensure cultural validity in a Chichewa speaking culture, the translated tools were checked by a Malawian Mental Health Clinician, and pre-tested amongst a group of Malawian adolescents. Translation of the English concepts to Chichewa was straightforward for both tools. The process did identify several items that needed clarification, for example, CDI-II-S #12: in Chichewa “feeling” translated directly to “kumva” which could also mean “hearing.” Therefore, we opted to use the word “kusungulumwa” meaning, “feeling alone.” BDI-II #19: “weight loss” would translate to “decreasing weight” in Chichewa, which would be too long and unclear. Therefore, we used instead “kutsika kwa sikelo” meaning “decrease in scale” since weight in Malawi is commonly referred to as “scale=sikelo.”

CDRS-R is the most widely used rating tool for the assessment of depressive symptoms in children and adolescents, particularly in international research trials [40–42]. It is a clinician interview hand-rated instrument that covers 17 symptom areas of depression and can be used to both diagnose and measure treatment response to depression [36,38,43]. The CDRS-R was based on the adult Hamilton Depression Rating Scale and can be administered in 15–20 minutes [39,43]. It rates 14 of 17 items from 1 to 7 while the remaining three items are rated 1–5 [39]. The rating of the items is between 1 (=no difficulties) and 5, or 1 and 7 (7=clinically significant difficulties) summing up to a total potential raw score of 113 [41]. A child's non-verbal behaviour is rated by the observer for items 15–17 [39]. A raw score of ≥30 with a T-score of ≥55 has been proposed to be indicative of depression [39].

In sub-Saharan Africa, there is scarcity of research in child mental health and likewise paucity of data concerning the psychometric properties of the CDRS-R. However, the developers of the tool report internal consistency (Cronbach's alpha) of 0.85, inter-rater reliabilities of 0.92–0.96, test–retest reliability of 0.80 as well as evidence of extensive convergent validity, moderate concurrent validity, discriminative validity and predictive validity [43]. Many other studies in Asia, Europe and America have demonstrated excellent psychometric properties of the CDRS-R [36,39,40,41,43].

In Malawi, there is currently only one psychiatrist working in the public sector. Therefore, although published studies utilizing the CDRS-R in Malawi are lacking, given the strong evidence supporting the use of CDRS-R internationally, in lieu of a psychiatric interview to diagnose depression, we utilized the validated English version of CDRS-R to help assess convergent validity of the BDI-II and CDI-II-S. To help ensure optimal administration, only clinicians with mental health training and over two years of experience in adolescent HIV care, were selected to undergo one week training in the administration of the CDRS-R. The training involved discussing and agreeing upon locally equivalent words for terminology. The post training competency assessment included observed administration of the CDRS-R to ensure standardized administration and use of culturally accepted vocabulary. Interviewers received on-going supervision by a qualified mental health professional with expertise in adolescent depression.

### Procedure

Participants’ interviews were conducted between January and August 2012. Consents/assents were obtained from caregivers and adolescents. Adolescents first completed the CDI-II-S and then the BDI-II in a private room. Finally, trained clinicians, who were blinded to the results of the CDI-II-S and BDI-II, administered the CDRS-R. All participants who were determined as having depression were promptly referred to a Mental Health Clinical Officer and Psychosocial Counsellor on site for further assessment and expert management.

### Data analysis

Descriptive statistics, such as mean and standard deviation (SD) for continuous variables, and frequency and proportion for categorical variables, were calculated. We used CDRS-R T-score, BDI-II total raw score and CDI-II-S T-score in the analysis. The CDRS-R was used as gold standard to classify depression. The prevalence of depression was calculated by the proportion of subjects with CDRS-R score ≥55. Chi-square test was used to compare the prevalence of depression between genders. Sensitivity, specificity, and positive and negative predictive values for various cut-offs were calculated to determine the optimal screening as well as diagnostic threshold with receiver operating characteristics (ROC) analysis.

Optimal cut-offs were determined first by identifying the point that gave the smallest distance from the ROC curve to the upper left corner of the graph. This point minimizes the sum of squares of false negative (1-sensitvity) and false positive (1-specificity). This cut-off gave the best discrimination between cases and non-cases. The second approach was to find a point that maximizes both sensitivity and specificity by determining the point at which sensitivity approximates specificity. The area under the curve (AUC) was calculated to determine the tools’ diagnostic ability; AUC of 1.00 indicating perfect diagnostic ability. The SAS logistic procedure estimated the AUC using the trapezoid rule and compared the AUCs using the Chi-square test according to DeLong [44].

Since variances between items were widely spread, internal consistency was measured by standardized Cronbach's alpha coefficient. Measurements were not normally distributed; therefore, the Spearman's correlation coefficient was used to measure the correlation between the two tools and the clinical interview. For all statistical tests, a two sided *p*<0.05 was considered significant. Data was analyzed using SAS version 9.3 (SAS Institute, Cary, NC).

## Results

The two clinics provided a list of 695 potential participants. Of these, 102 could not be contacted due to death, loss to follow-up, lack of contact information or transfer to another facility. Of those contacted, 11 were unable to participate due to disability. Of the 582 eligible participants, 97% (562) consented/assented and enrolled in the study.

### Descriptive characteristics

Table 1 describes the socio-demographic characteristics of the adolescent study participants.

**Table 1:** Socio-demographic characteristics of the study participants

Variable	*n* (%) *N*=562
Age (mean±SD)	14.5±2.0
Sex	
Female	315 (56.1)
Male	247 (44.0)
Residential location	
Urban	369 (65.7)
Peri-urban	93 (16.5)
Rural	100 (17.8)
Education status	
Primary School	402 (71.5)
Secondary School	153 (27.2)
Post-Secondary School	4 (0.7)
Not at School	3 (0.5)

The mean scores (±SD) were 11.9±7.9 for BDI-II and 51.6±10.2 for CDI-II-S. Females had significantly higher mean BDI-II than males (12.8±8.1 vs. 10.9±7.5, *p*=0.005); CDI-II-S scores were similar between genders (*p*=0.56).

### Prevalence of depression

The prevalence of depression as measured by the CDRS-R was 18.9% (106/562): 21.6% (68/315) in females and 15.4% (38/247) in males. Although this finding approached significance (*p*=0.06), the difference in mean CDRS-R scores was significant, 48.7±7.2 (females) and 47.2±6.7 (males), *p*=0.013. Using the CDRS-R (score of >2 on question #13), suicidal ideation was expressed by 3.0% (17), and using BDI-II (score of >0 on question #9) by 7.1% (40) (*p<*0.001).

### Psychometric evaluation of the BDI-II and CDI-II-S

#### Diagnostic accuracy

**Figure 1:**
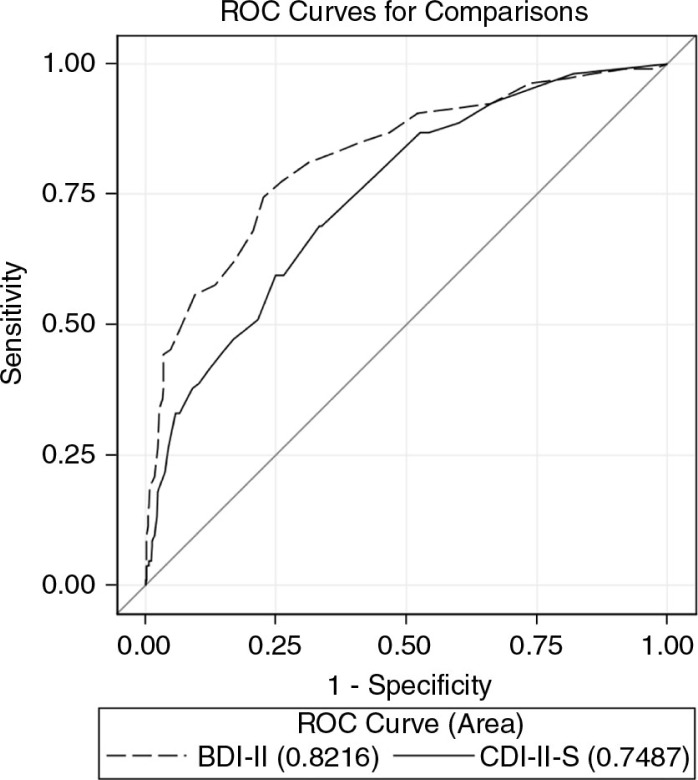
Receiver operating characteristic (ROC) curve for the BDI-II and CDI-II-S as compared to the CDRS-R.

Figure 1 demonstrates the ROC curves for the two tools as compared to the CDRS-R. The AUC of the BDI-II 0.82 (95% CI 0.77–0.87) was significantly greater than the AUC of the CDI-II-S 0.75 (95% CI 0.70–0.80) (*p*=0.003). By gender, the AUCs for the BDI-II were 0.88 (males) and 0.78 (females), and for CDI-II-S were 0.85 (males) and 0.69 (females). The difference between the scales was significant among females (*p*=0.007) but not among males (*p*=0.35).

**Figure 2:**
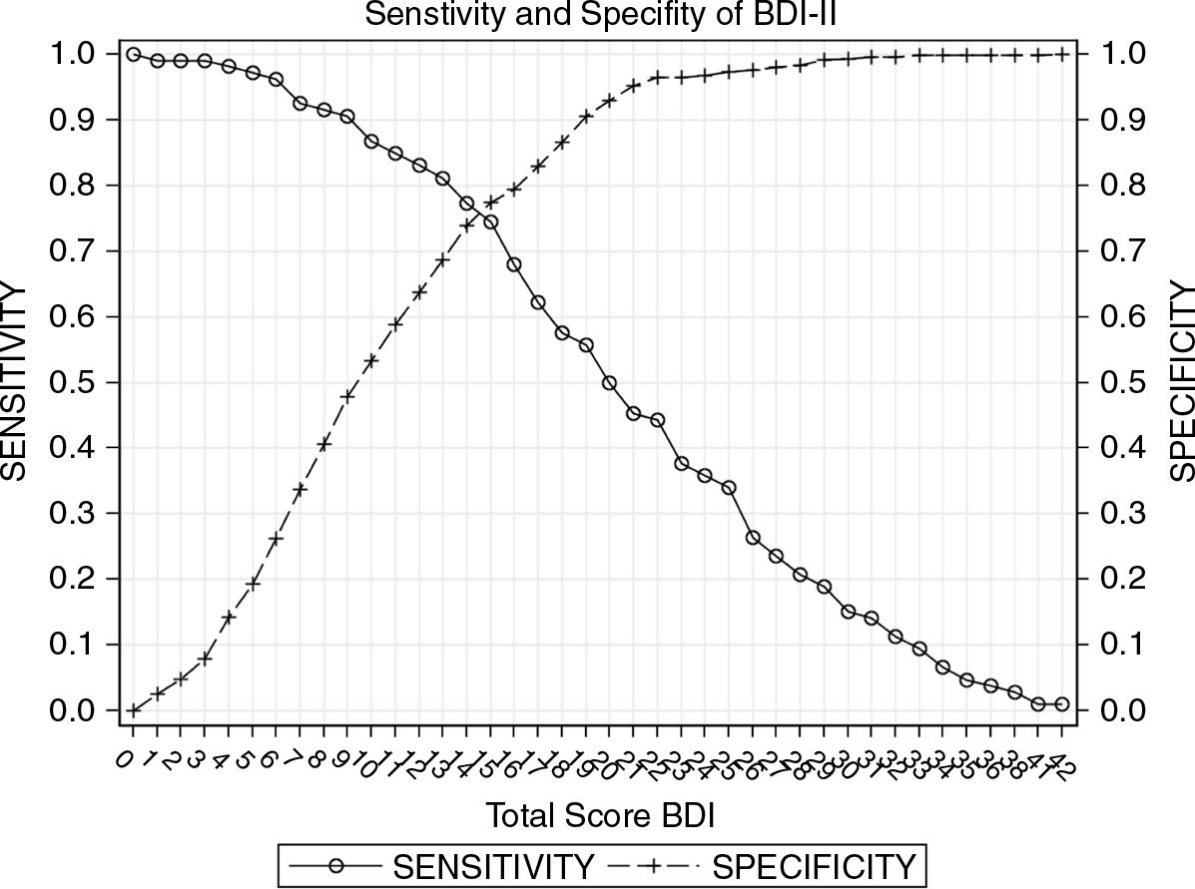
Sensitivity and specificity of the BDI-II.

**Figure 3:**
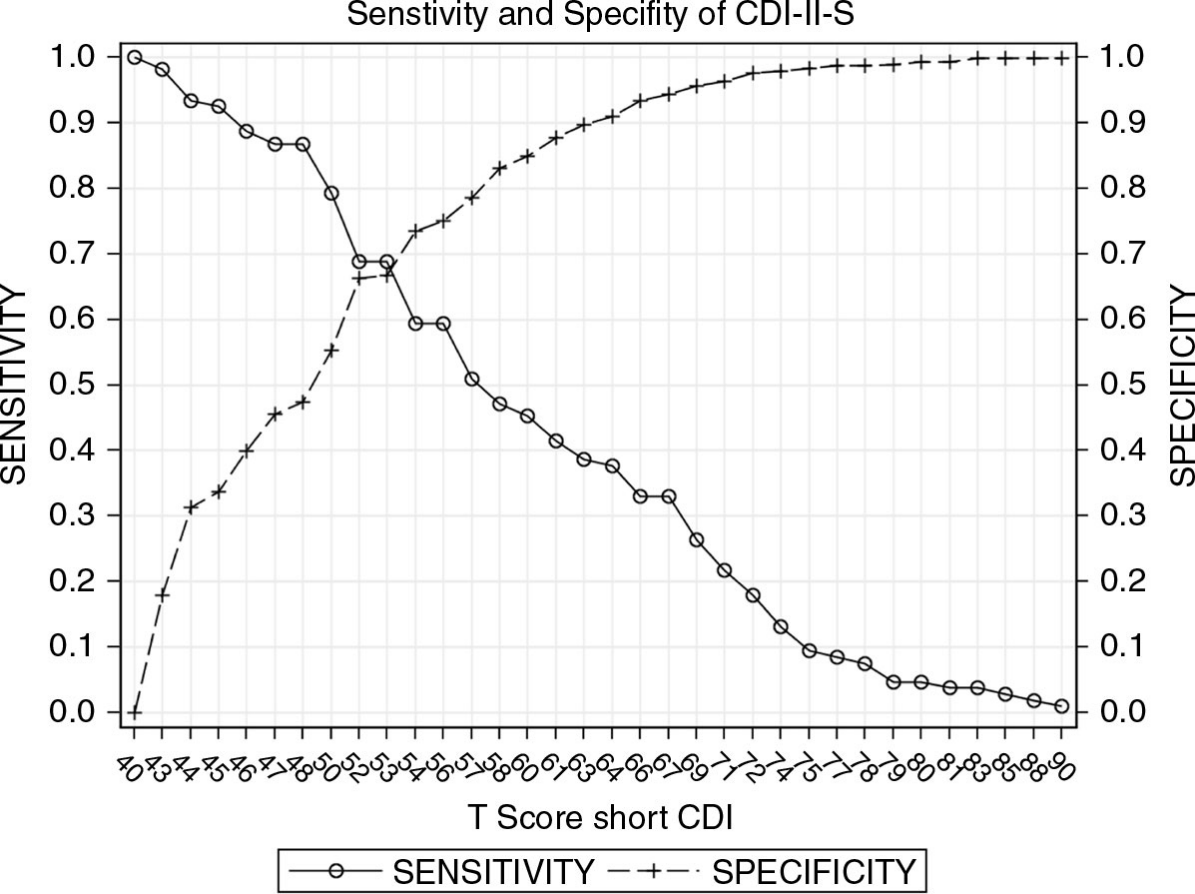
Sensitivity and specificity of the CDI-II-S.

The sensitivity, specificity, positive predictive value, and negative predictive value at various cut-off scores for both tools are shown in Tables 2 and 3 and Figures 2 and 3.

**Table 2:** Psychometric properties of the BDI-II in screening for depression in HIV-positive Malawian adolescents

Cut-off BDI-II (raw score)	Specificity	Sensitivity	PPV	NPV
11	0.588	0.849	0.324	0.944
12	0.638	0.830	0.348	0.942
13	0.686	0.811	0.376	0.940
14	0.739	0.774	0.408	0.934
**15**	**0.774**	**0.745**	**0.434**	**0.929**
16	0.794	0.679	0.434	0.914
17	0.829	0.623	0.458	0.904
18	0.866	0.575	0.500	0.898
19	0.906	0.557	0.578	0.898
20	0.930	0.500	0.624	0.889

**Table 3:** Psychometric properties of the CDI-II-S in screening for depression in HIV-positive Malawian adolescents

Cut-off CDI-II-S (T-score)	Specificity	Sensitivity	PPV	NPV
47	0.456	0.868	0.271	0.937
48	0.474	0.868	0.277	0.939
50	0.553	0.792	0.292	0.920
52	0.662	0.689	0.322	0.901
**53**	**0.667**	**0.689**	**0.324**	**0.902**
54	0.735	0.594	0.342	0.886
56	0.750	0.594	0.356	0.888
57	0.785	0.509	0.355	0.873
58	0.831	0.472	0.394	0.871

For the BDI-II, a cut-off of 15 gives both the shortest distance from the ROC curve to the upper left corner of the graph as well as the point where specificity approximates sensitivity. This cut-off remains consistent for both genders. A score of ≥13 achieved sensitivity of >80% and, therefore, may be ideal to screen for cases, whereas a score of ≥17 had a specificity of >80%, making it appropriate for identifying non-cases (diagnosis).

For the CDI-II-S, a cut-off of 53 gives both the shortest distance from the ROC curve to the upper left corner of the graph as well as the point where specificity approximates sensitivity. Cut-offs differ slightly by gender: 52–53 (females) and 54–57 (males). Specificity approximates sensitivity at 52 (females) and 56 (males). The shortest distance from ROC curve to the upper left corner of the graph was 53 (females) and 57 (males). Overall, a score of ≥ 48 achieved sensitivity of >80% and, therefore, may be ideal as a screening cut-off, whereas a score of ≥ 58 had a specificity of >90%, making it appropriate for diagnostic use.

#### Internal consistency and validity

The Cronbach's alpha for the BDI-II was 0.80 indicating strong internal consistency. The Cronbach's alpha for the CDI-II-S was 0.66. The convergent validity between the BDI-II and CDI-II-S as calculated by the Spearman correlation was 0.54 (*p*<0.001). The correlation between the BDI-II and CDRS-R was 0.42 (*p*<0.001). The correlation between the CDI-II-S and CDRS-R was 0.37 (*p*<0.001). Similar internal consistency (BDI-II: 0.80 vs. 0.80; CDI-II-S: 0.65 vs. 0.66) and medium correlation (BDI-II and CDRS-R: 0.38 vs. 0.45; CDI-II-S and CDRS-R: 0.31 vs. 0.44) were found by female vs. male gender.

## Discussion

To our knowledge, this is the first study to estimate the prevalence of depression amongst HIV-positive Malawian youth as well as the first study to evaluate the psychometric properties of the BDI-II and CDI-II-S for depression screening in this population. Our findings suggest that the prevalence of depression is high and that the BDI-II can be used to screen for depression amongst this population. The internal consistency of the CDI-II-S as measured by Cronbach's alpha was lower than the 0.73–0.91 established in American samples across all versions of the CDI-II [29]. However, the internal consistency is similar to what was reported in Tanzania amongst orphans (0.67) [13]. The relatively lower internal consistency estimates suggest that additional modifications may need to be made to the CDI-II-S for use in this population. In all, the BDI-II had superior internal consistency and AUC, as well as better concordance with the clinical interview. Furthermore, our study suggests a score of ≥13 in BDI would be appropriate as a screening cut-off score, whereas a score of ≥17 would be more appropriate for diagnostic use.

Due to the paucity of studies in HIV-positive adolescents, different age ranges and diverse measures, it is difficult to make prevalence comparisons. However, our prevalence of 18.9% using the CDRS-R was similar to the 17.8% prevalence found amongst HIV-positive children, 6–18 years of age in Kenya using the Mini International Neuropsychiatric Interview for Major Depressive Episode (MINI-kid) [14], and 18.3% found in Malaysia (Diagnostic and Statistical Manual of Mental Disorders, Fourth Edition and MINI-kid) [37]. Since the CDRS-R, unlike the MINI-kid, was not designed to identify other mental disorders such as bipolar and anxiety disorders, we were unable to report on other potentially co-morbid mental health conditions. Studies in both HIV-positive and uninfected adolescents and adults have demonstrated that females show higher rates of depression compared to males [22,23,45,46]. In our study, the difference in prevalence of depression between genders appeared to be considerable, and we found a statistically significant difference in mean CDRS-R scores between genders. Interestingly, suicidal ideation as expressed by the BDI-II was higher than that found by the CDRS-R.

It is possible that having a more highly trained and sympathetic interviewer could encourage a participant to be more forthcoming in reporting symptoms. However, typically, there is higher endorsement of all psychopathology on self-report screening measures like the BDI-II versus interview-based measures [38]. The youth in this study may have felt more comfortable answering certain questions by self-report versus interview.

There were several limitations to the study. Given the extremely limited number of qualified psychiatrists in Malawi, we opted for assessment of convergent validity with the CDRS-R, in lieu of the gold standard diagnosis of depression via psychiatric interview. Results demonstrated a medium correlation between the BDI-II and the CDRS-R probably reflecting a method effect (self-report vs. interview-based). More research using interview-based tools alongside self-report measures is needed to clarify these results. In addition, we cannot definitively conclude that our prevalence estimate is representative of all HIV-positive adolescents in Malawi. However, the Baylor COE is the largest referral centre for paediatric HIV in Malawi, with patients coming from a wide geographic area. Zomba Central Hospital ART Clinic in southern Malawi is one of two referral centres for patients from the southern region. Therefore, sampling from these centres should provide a fairly reliable estimate of prevalence of depression amongst HIV-positive Malawian youth.

As the study did not include a control group and national statistics on depression prevalence in Malawian adolescents are lacking, we were unable to conclude whether our prevalence estimate is higher than that of non-HIV-positive adolescents in Malawi. In addition, caution should be exercised in generalizing the results, including use of the tools to adolescents without HIV infection. However, the high prevalence of depression amongst Malawian adolescents living with HIV highlights a significant mental health need, and raises a concern regarding how these needs will be met in a resource-limited setting.

The strength of our study is that it is the first epidemiological study to assess the prevalence of depression in HIV-positive youth in Malawi, and the first to use two self-report measures and a semi-structured diagnostic instrument. In addition, to our knowledge, this is the largest mental health prevalence study done in HIV-positive youth in Africa.

## Conclusions

Despite the limitations, this study has demonstrated that the BDI-II has sound psychometric properties in an outpatient setting among HIV-positive adolescents in Malawi. Our study also supports the use of the BDI-II as a viable measure for identifying possible cases of depression amongst this population and, in fact, may be easier to administer as compared to an interview-based tool for assessing depression. The CDI-II-S can be used to screen for depressive symptoms. However, the low internal consistency estimates suggest that modifications may need to be made. Importantly, our study demonstrates a high prevalence of depression amongst HIV-positive Malawian youth and underscores the need for the development of comprehensive services for HIV-positive adolescents. Further research is needed to explore factors contributing to and protective of depression in adolescents living with HIV/AIDS. Interventional studies are also needed to determine the most efficacious treatments.

## References

[1] UNICEF (2011). Opportunity in Crisis preventing HIV from early adolescence to young adulthood.

[2] UNAIDS (2011). Securing the future today synthesis of strategic information on HIV and young people.

[3] Jaspan HB, Li R, Johnson L, Bekker LG (2009). The emerging need for adolescent focused HIV care in South Africa. South Afr J HIV Med.

[4] Cowan F, Pettifor A (2009). HIV and adolescents in Sub-Saharan Africa. Curr Opin HIV/AIDS.

[5] WHO (2001). The World Health Report 2001: mental health: new understanding, new hope. Report.

[6] WHO (2008). Global burden of disease, 2004 update.

[7] Weissman MM, Bland RC, Canino GJ, Faravelli C, Greenwald S, Hwu H (1996). Cross national epidemiology of major depression and bipolar disorder. JAMA.

[8] HIV/AIDS and depression in Africa (2009). an international mental health awareness packet from the WFMH Africa initiative on mental health & HIV/AIDS. http://www.wfmh.org.

[9] Gupta R, Dandu M, Packel L, Rutherford G, Leiter K, Phaladze N (2010). Depression and HIV in Botswana: a population-based study on gender-specific socioeconomic and behavioral correlates. PLoS One.

[10] Nakasujja N, Skolasky RL, Musisi S, Allebeck P, Robertson K, Ronald A (2010). Depression symptoms and cognitive function among individuals with advanced HIV infection initiating HAART in Uganda. BMC Psychiatry.

[11] Nduna MJ, Jewkes RK, Dunkle KL, Jama Shai NP, Colman I (2010). Associations between depressive symptoms, sexual behaviour and relationship characteristics: a prospective cohort study of young women and men in the Eastern Cape, South Africa. J Int AIDS Soc.

[12] Masulani-Mwale C (2006). The prevalence of psychological distress and associated factors among people living with AIDS attending antiretroviral therapy clinics in Mzuzu.

[13] Traube D, Dukay V, Kaaya S, Reyes H, Mellins C (2010). Cross-cultural adaptation of the Child Depression Inventory for use in Tanzania with children affected by HIV. Vulnerable Child Youth Stud.

[14] Kamau JW, Kuria W, Mathai M, Atwoli L, Kangethe R (2012). Psychiatric morbidity among HIV-infected children and adolescents in a resource-poor Kenyan urban community. AIDS Care.

[15] Farinpour R, Miller EN, Satz P, Selnes OA, Cohen BA, Becker JT (2003). Psychosocial risk factors of HIV morbidity and mortality: findings from the Multicenter AIDS Cohort Study (MACS). J Clin Exp Neuropsychol.

[16] Cruess DG, Petitto JM, Leserman J, Douglas SD, Gettes DR, Ten Have TR (2003). Depression and HIV infection: impact on immune function and disease progression. CNS Spectrums.

[17] Treisman GJ, Angelino AF, Hutton HE (2001). Psychiatric issues in the management of patients with HIV infection. JAMA.

[18] Holzemer WL, Corless IB, Nokes KM, Turner JG, Brown MA, Powell-Cope GM (1999). Predictors of self-reported adherence in persons living with HIV disease. AIDS Patient Care STDs.

[19] Gonzalez JS, Batchelder AW, Psaros C, Safren SA (2011). Depression and HIV/AIDS treatment nonadherence: a review and meta-analysis. J Acquir Immune Defic Syndr.

[20] Rubin AG, Gold MA, Primack BA (2009). Associations between depressive symptoms and sexual risk behavior in a diverse sample of female adolescents. J Pediatr Adolesc Gynecol.

[21] Stewart RC, Kauye F, Umar E, Vokhiwa M, Bunn J, Fitzgerald M (2009). Validation of a Chichewa version of the self-reporting questionnaire (SRQ) as a brief screening measure for maternal depressive disorder in Malawi, Africa. J Affect Disord.

[22] Adewuya AO, Ola BA, Aloba OO (2007). Prevalence of major depressive disorders and a validation of the Beck Depression Inventory among Nigerian adolescents. Eur Child Adolesc Psychiatr.

[23] Monahan PO, Shacham E, Reece M, Kroenke K, Ong'or WO, Omollo O (2009). Validity/reliability of PHQ-9 and PHQ-2 depression scales among adults living with HIV/AIDS in western Kenya. J Gen Intern Med.

[24] Reda AA (2011). Reliability and validity of the Ethiopian version of the hospital anxiety and depression scale (HADS) in HIV infected patients. PLoS One.

[25] Bass JK, Bolton PA, Murray LK (2007). Do not forget culture when studying mental health. Lancet.

[26] Beck AT, Steer RA, Brown GK (2006). RCMAR measurement tools: Beck Depression Inventory – 2nd Edition (BDI-II). http://academicdepartments.musc.edu/family_medicine/rcmar/beck.htm.

[27] Frey RJ (2003). Child Depression Inventory. Gale Encyclopedia of Mental Disorders. Encyclopedia.com.

[28] Kovacs M Children's Depression Inventory: Short Version (CDI:S). http://www.nzcer.org.nz/sites/default/files/CDI%20Profile%20Report%20(Short).pdf.

[29] Politi D (2012). Education and clinical assessment consultant Children's Depression Inventory 2nd Edition (CDI-2): introduction and application (Multi-Health Systems (MHS) [Internet]. http://www.mspaonline.net/CDI%202%20Handouts%201-12.pdf.

[30] Government of Malawi Ministry of Health (2011). Quarterly HIV Programme Report: HIV testing and counseling, prevention of mother to child transmission, antiretroviral yherapy.

[31] Mkalira K (2009). The prevalence of depression and its correlates among orphans within Bangwe Township, Blantyre [Prevalence survey] [thesis].

[32] Kovacs M (2004). Children's Depression Inventory (CDI). http://www.psychassessments.com.au/products/22/prod22_report1.pdf.

[33] Angus HT (2012). Childhood depression revisited: indicators, normative tests and clinical course. J Can Acad Child Adolesc Psychiatry.

[34] Children's Depression Inventory – Second Edition (CDI-2) [Internet] Toronto. Multi Health Systems Inc (2004). http://www.mhs.com/product.aspx?gr=edu&prod=cdi2&id=overview#scales.

[35] Rivera CL, Bernal G, Rossello J (2005). The Children Depression Inventory (CDI) and the Beck Depression Inventory (BDI): their validity as screening measures for major depression in a group of Puerto Rican adolescents. Int J Clin Helath Psychol.

[36] Zalsman G, Misgav S, Sommerfeld E, Kohn Y, Brunstein-Klomek A, Diller R (2011). Children's Depression Inventory (CDI) and the Children's Depression Rating Scale-Revised (CDRS-R): reliability of the Hebrew version. Int J Adolesc Med Health.

[37] Rosliwati MY, Rohayah H, Jamil BYM, Zaharah S (2008). Validation of the Malay version of Children Depression Inventory (CDI) among children and adolescents attending outpatient Clinics in Kota Bharu, Kelantan. Malays J Psychiatr.

[38] Cusin C, Yang H, Yeung A, Fava M (2010). Rating Scales for Depression. Rating Scales foe Depression.

[39] Basker MMR, Russell PSS, Russell S, Moses PD (2010). Validation of the children's depression rating scale– revised for adolescents in primary-care pediatric use in India. Indian J Med Sci.

[40] Mayes TL, Bernstein IH, Haley CL, Kennard BD, Emslie GJ (2010). Psychometric properties of the Children's. Depression Rating Scale-Revised in adolescents. J Child Adolesc Psychopharmacol.

[41] Plener PL, Grieb J, Sproeber N, Straub J, Schneider A, Keller F, Koelch MG (2012). Convergence of children's depression rating scale-revised scores and clinical diagnosis in rating adolescent depressive symptomatology. Ment Illness.

[42] Poznanski EO, Mokros HB (1996). Children's Depression Rating Scale, Revised (CDRS-R).

[43] Poznanski E, Cook SC, Carroll BJ (2013). Measure profile: Children's Depression Rating Scale (CDRS).

[44] DeLong ER, DeLong DM, Clarke-Pearson DL (1988). Comparing the areas under two or more correlated receiver operating characteristic curves: a nonparametric approach. Biometrics.

[45] Maharaj RG, Alli F, Cumberbatch K, Laloo P, Mohammed S, Ramesar A (2008). Depression among adolescents, aged 13–19 years, attending secondary schools in Trinidad prevalence and associated factors. West Indian Med J.

[46] Bodur S, Kücükkendirci H (2009). Prevalence of depressive symptoms in Turkish adolescents. Eur J Gen Med.

